# Enquiry analysis and user opinion of the Drugs in Breastmilk Helpline: a prospective study

**DOI:** 10.1186/1746-4358-7-6

**Published:** 2012-05-02

**Authors:** Paul M Rutter, Wendy Jones

**Affiliations:** 1 Department of Pharmacy, School of Applied Sciences, University of Wolverhamptonm, Wulfruna Street, Wolverhampton, WV1 1SB, UK; 2 The Breastfeeding Network Drugs in Breastmilk Helpline, The Breastfeeding Network, PO Box 11126, Paisley, PA2 8YB, UK

**Keywords:** Medication, Breastmilk, Helpline, Evaluation, Breastfeeding, Health personnel

## Abstract

**Background:**

Since breastfeeding is universally recognised as the ideal way to feed infants, it is understandable, and at times inevitable, that breastfeeding mothers will want, or be required, to take medication. To meet the information demands of breastfeeding mothers and healthcare professionals, a UK charity, The Breastfeeding Network, established a free telephone helpline to answer queries on medicines in breastmilk. This study reports on the enquiries received by the Drugs in Breastmilk Helpline and user opinion of the service.

**Methods:**

All enquirers to the Helpline between December 2010 and January 2011 were asked if they could be contacted in 2 to 4 weeks to provide more information on their experience of using the service. A combination of telephone semi-structured interviews and email surveys were used depending on whether the enquiry originated via telephone or email.

**Results:**

Information was gained from 101 participants; 77 women and 24 healthcare professionals. Women reported high levels of service satisfaction (94%, n = 72/77) and healthcare professionals found the information provided useful (92%, n = 22/24). Women used the service for reassurance or because they had received conflicting information or distrusted healthcare professional advice. Healthcare professionals often could not answer questions or took a cautious approach to recommendation (i.e. advised avoidance of medicines whilst breastfeeding); this was often at odds to advice given by staff from the Helpline. Healthcare professionals did not routinely access resources to answer questions, but when they did, showed a lack of confidence in data interpretation.

**Conclusions:**

The Breastfeeding Networks’ Drugs in Breastmilk Helpline provides an important service to breastfeeding women and healthcare staff to make informed decisions on medicine taking whilst breastfeeding. Healthcare professional uncertainty and incorrect advice given to breastfeeding women suggests that healthcare professional education needs improving and that greater use of specialist services should be encouraged.

## Background

It is widely acknowledged that breastfeeding provides the ideal nutrition for infants, with authorities worldwide recommending exclusive breastfeeding for the first six months for children living in developed countries [1,2]. Despite such endorsements, studies have shown that physician knowledge on breastfeeding is far from ideal [3-8], as they receive relatively little formal training on breastfeeding [5], and often fail to follow evidence-based medicine (EBM) guidelines [9]. However, it can be difficult for physicians to keep up-to-date with the high volume of constantly changing, sometimes conflicting, information. It has been suggested that a physician needs to read 17 articles each day to keep up with current medical literature, yet they have limited time to assess the information they receive [10]. Additionally, studies have shown that access to EBM resources is variable [11-16], and even when resources are available, the ability of physicians to locate relevant information and assess its validity appears limited [11,12,17].

It is therefore unsurprising that physicians may turn to specialist information services for help. Such services include, UKMi (a UK National Health Service [NHS] funded medicines information service that has specialist centres taking medicine calls on pregnancy and breastfeeding) [18] and the Motherisk Program (a Canadian-based teratogen information service) [19]. These services tend to be aimed at healthcare professionals, who report high levels of satisfaction with the information provided [20,21]. Equivalent information services for consumers are limited, but the Breastfeeding Network (BfN) (a UK-based charity established in 1997) aims to be an independent source of support and information for breastfeeding women and others, including healthcare professionals [22]. One of the BfN services is the provision of ‘Supporterline’; a service where information and support is provided to mothers who are breastfeeding that is complementary to that of health visitors (a health visitor is a UK qualified nurse whose role promotes health in the whole community, particularly involved with families who have children under five), midwives and other healthcare professionals. In 2006 the Supporterline took over 20,000 calls, many specifically related to medicine use. Consequently, in 2007 a national 0800 (free) telephone service for medicine-related enquiries was established, The Drugs in Breastmilk Helpline, which has seen year-on-year increases in enquiry numbers (e.g. 576 in 2007, which had risen to 2215 in 2010). To cope with this increased demand, the service now operates seven days a week and when not manned takes calls via an answer machine. The majority of enquiries are answered by a pharmacist, with support from a trained BfN worker. The Helpline has access to standard medical reference texts (e.g. The British National Formulary [23]) and specialist breastfeeding references, for example Hale's *Medications and Mother’s Milk*[24], LactMed (an online database from The National Library of Medicine) [25], Briggs et al's *Drugs in Pregnancy and Lactation*[26] and *Martindale The Extra Pharmacopoeia*[27].

Given the growth in user numbers, the aim of this study was to assess consumer and healthcare professional opinion, analyse the enquiries taken and to ascertain how information provided was used.

## Methods

Enquirer opinion was surveyed via a structured telephone interview or self-administered survey. A sample size calculator [28] indicated that 90 participants were required to achieve 95% confidence (with a 10% margin for error) so that the study results reflect the results expected from the total enquirer population to the Helpline (in 2009, n = 1388). Analysis of workload data and factoring in a 40% drop-out rate indicated that the study duration would be approximately two months. Participant recruitment began in December 2010.

Enquiries were generated either by telephone call or email. All enquirers who contacted the service were eligible to be enrolled in to the study, provided they were English speakers. For telephone enquiries, prior to the BfN call handler concluding the conversation, the enquirer was informed of the study and asked if they would be happy to be contacted in 2 to 4 weeks time to discuss the call further. For those people who agreed to be contacted, their details, along with the enquiry and answer provided by the Helpline, were sent to researchers at the University of Wolverhampton. Each caller was then telephoned and interviewed. Interviews followed a semi-structured format and took approximately 10 min to complete.

For email replies, information relating to the study was included at the bottom of the written emailed reply, along with an invitation to complete the self-administered survey. Return of the email was taken as the person giving consent to participate. Questions used for both the interviews and surveys were drawn and adapted from UKMi surveys used to quality assure their service.

Questions asked differed depending on whether the enquirer was a member of the public or a healthcare professional. However, the questions asked to each respective group were the same for both the telephone interview and self-administered survey. Data collected fell broadly into three sections: demographic information; evaluation of the Helpline; and usefulness of the information. Questions consisted of a mixture of open, closed and semantic differential scales (e.g. 5 point Likert scales). Prior to data collection starting, a two-week pilot study was undertaken on the study population to determine the data collection tools’ content validity and reliability. Feedback from the pilot study showed that no changes were required.

Research ethics committee approval was granted by The Behavioural Sciences Research Ethics Committee at Wolverhampton University. NHS ethics approval was not required as the work was assessed as evaluation and not research.

## Data analysis

Responses were collated, stored and analysed using survey software (SNAP for Windows) and spreadsheets (Microsoft Excel 2003 for Windows). Data entry were performed by two research assistants and the validity of data entry assured by a member of the research team (PR) who was not involved in data entry. PR undertook random checks of the data (equating to approximately 20% of all data). Simple descriptive statistics were used to summarise respondents’ data. Free text answers were reviewed and categorised by PR. Data were managed using Excel and common themes generated that were agreed upon by both authors.

## Results

One hundred and forty-two enquirers were contacted. On follow up, twenty-one declined to participate and twenty were lost, leaving 101 replies for analysis: 77 from women (64 telephone calls and 13 emails); and 24 from healthcare professionals (10 telephone calls and 14 emails).

### Data from breastfeeding women

#### Demographic characteristics

Most women were under the age of forty (88%, n = 68/77), had either 1 or 2 children (87%, n = 67/77), undertook formal education beyond A levels (84%, n = 63/75) (i.e. further education colleges or university) and were Caucasian (92%, n = 70/76). No calls were received from any person under the age of twenty-five. Demographic data are shown in Table 1.

**Table 1:** Characteristics of participants

**Demographic characteristics of the breastfeeding women enquirers**			
		**n**	**%**
Age (n = 77)			
	25-29	18	23
	30-34	29	38
	35-39	21	27
	over 40	9	12
Which child is this in your family? (n = 77)			
	first	46	60
	second	21	27
	third	8	10
	fourth	1	1
	fifth	1	1
Ethnic origin (n = 76)			
	white	70	92
	mixed	2	3
	Asian/Asian British	3	4
	other	1	1
School leaving age (n = 75)			
	16 or under	8	11
	17	4	5
	18	12	16
	19 or over	51	68

**Healthcare professional occupation (n = 24)**			
		**n**	**%**
	Infant feeding co-ordinator	8	33
	Health visitor	6	25
	Midwife	4	17
	Physician	1	4
	Pharmacist	1	4
	Community staff nurse	1	4
	Community breastfeeding leader	1	4
	Hospital nurse specialising in lactation	1	4
	Not stated	1	4

#### Breastfeeding women’s opinion on the helpline

Their experience with the service was very positive. Seventy (91%) women were either ‘very satisfied’ or ‘satisfied’ with how quickly their call was answered and stated that the answer they received provided sufficient information to enable them to make a decision regarding their enquiry (90%, n = 69/77). These findings are reflected in the level of satisfaction noted, where 72 (94%) were either ‘very satisfied’ or ‘satisfied’ with the overall service offered (Table 2). Only three women felt that phoning the Helpline had not helped them. Analysis of these three calls showed that two involved complex calls, involving persistent nipple and breast pain that were being managed unsatisfactorily by their physician. The third case involved use of a topical nasal decongestant whilst breastfeeding. Advice given was that the woman could use the product, so it is unclear as to why this person said the advice did not provide enough information to make a decision.

**Table 2:** Questions asked of breastfeeding women and healthcare professionals

	**Healthcare professionals (n = 24)**		**Breastfeeding women (n = 77)**	
	**n**	**%**	**n**	**%**
**How did you find out about the telephone number?**				
Birth to Five	0	0	12	16
given it by someone	6	26	23	30
internet	8	35	27	35
leaflet	8	35	16	21
other	1	4	0	0
**Have you called the Drugs in Breastmilk Helpline before?**				
Yes	17	71	13	17
No	7	30	64	83
**Would you recommend this service to others?**				
Yes	22	92	77	100
No	2	9	0	0
**Did the information provide you with enough information to make a decision?**				
Completely	20	87	69	90
Partly	3	13	5	7
Not at all	0	0	3	4
**How satisfied were you with the response to your query, with respect to speed?**				
Very satisfied	N/A	N/A	64	83
Satisfied	N/A	N/A	6	8
Neither	N/A	N/A	5	7
Dissatisfied	N/A	N/A	2	3
Very dissatisfied	N/A	N/A	0	0
**Overall, how satisfied were you with the service?**				
Very satisfied	N/A	N/A	69	90
Satisfied	N/A	N/A	3	4
Neither	N/A	N/A	4	5
Dissatisfied	N/A	N/A	1	1
Very dissatisfied	N/A	N/A	0	0

Despite these enquirers not having been given information to enable them to make a decision, they, as well as all other women, said they would recommend the service to other people (Table 2).

Most women (83%, n = 64) had not contacted the Helpline before and found out about the service primarily through the internet (35%, n = 27) or had been given the telephone number (30%, n = 23). Twelve (16%) had also been made aware by the ‘Birth to Five’ information booklet (an NHS information booklet distributed to all parents on the health, wellbeing and development of children from birth to five years old) (Table 2).

#### Content of enquiries

Seventy enquiries directly related to medicine taking and seven involved giving general advice (Table 3). Four of the latter involved issues of infant feeding whilst the mother was being treated with antifungal medication. Sixty-five enquiries involved the use of proposed medicines. These were predominantly to manage acute conditions, with self-management of minor illness (n = 11), anti-infectives (n = 14) and pain relief (n = 9) most frequently enquired about. Seventeen enquiries involved medicines that could be used for the management of long-term illness. These covered many medicine classes, with only medicines for depression (n = 4) and Crohn’s disease (n = 3) receiving more than one enquiry.

**Table 3:** Type of medicine-related enquiry received from participants

	**Proposed medicines**		**Currently taken medicinesN (%)**	**Advice givenn (%)**
	**For acute illness (n, %)**	**For chronic illness (n, %)**		
Healthcare professionals (n = 24)	13 (54%)	5 (21%)	2 (8%)	4 (16%)
Breastfeeding women (n = 77)*	50 (65%)	17 (22%)	5 (7%)	7 (9%)

* Two enquiries involved medicines for the treatment of proposed acute and chronic illness.

Five calls involved medicines that were currently being taken. Two concerned the effect medicines had on breastmilk (amitriptyline and penicillin), whilst the other three related to continuation of prescribed medication (asthma inhalers, azathioprine and propylthiouracil). The latter three enquirers had already spoken to a healthcare professional. In the calls involving azathioprine and propylthiouracil the women had been told by hospital physicians that they should not take the medicine, but this advice had been contradicted by pharmacists. In both instances the women wanted clarification on what to do. The Helpline advisor agreed with the pharmacist that the medicines could still be taken whilst breastfeeding. The call involving asthma inhalers centred on the woman wanting reassurance that the advice (to continue taking salbutamol and beclometasone inhalers) given by a practice nurse was correct, which it was.

The Helpline stated that of the 70 enquiries involving medicines, 68 could be taken whilst breastfeeding. All women followed the guidance given by the Helpline. In the two enquiries where the Helpline did not advise to take the medicine, both involved referral back to the physician on grounds of potential misdiagnosis.

**Figure 1:**
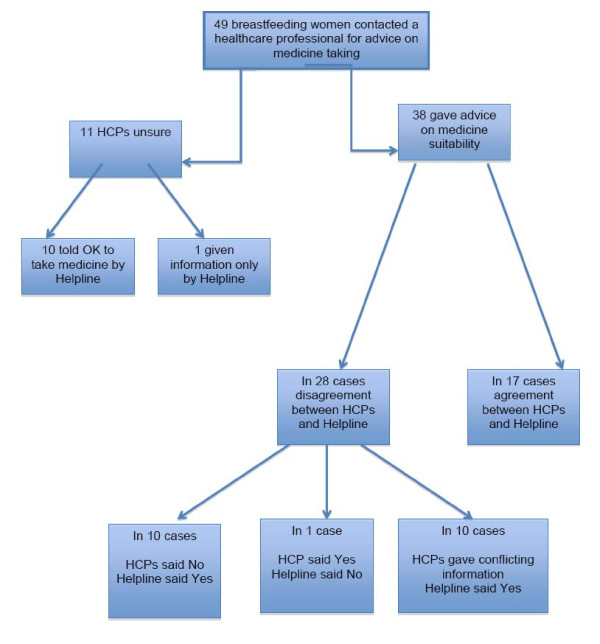
Handling of medicine-related enquiries from breastfeeding women by healthcare professionals (HCPs) and the Drugs in Breastmilk Helpline.

Fifty-three women contacted a healthcare professional before phoning the Helpline; 49 of these enquiries were about the suitability of taking a medicine (Figure 1). General practitioners (n = 12) and hospital doctors (n = 8) were most frequently asked for advice; other healthcare professionals were infrequently asked (pharmacists, n = 2; midwife n = 2; health visitor n = 2; practice nurse n = 1). In 10 instances women asked more than one healthcare professional for advice (in 7 cases two were asked, and in 3 cases three were asked). The need for reassurance, and receiving conflicting information were two common reasons why women called the Helpline. Typical comments were:

‘Conflicting information between different sources, wasn't sure which information was correct.’ (participant 70, telephone interview)

‘Conflicting information between the physician, pharmacist and drug information.’ (participant 40, telephone interview)

‘Instinctively I felt the drug would be fine, but just wanted to double check.’ (participant 5, email survey)

‘I had to know whether such an extended period of not breastfeeding was good for my baby. You were the only group offering advice tailored to breastfeeding mums.’ (participant 9, email survey)

Women also often spoke of distrust of the advice given by a healthcare professional or lack of confidence in their answer:

‘Lack of confidence in the doctor’s advice as he sounded confused and had to be reminded that I was breastfeeding.’ (participant 51, telephone interview)

‘Conflicting advice off the internet and didn't trust the consultant’s advice.’ (participant 31, telephone interview)

‘Pharmacist wasn't sure; wanted to see if ok to use while breastfeeding.’ (participant 33, telephone interview)

In 11 of the 49 enquiries, the healthcare professional (seven of whom were physicians) was unsure of the answer and unable to tell the person if the medicine could be taken whilst breastfeeding. For 10 of these 11 cases, staff from the Helpline, after consulting reference sources, were able to advise the women that the medicine could be safely taken. In the remaining enquiry, information was supplied to the woman but no definitive answer on whether the medicine could be taken was given. This enquiry was answered by the BfN worker and, following protocol, is only able to provide information and not advice on whether the medication can be taken.

Just 17 of the remaining 38 enquiries saw agreement between the healthcare professional and Helpline staff; in all these enquiries both parties agreed that the medicine could be taken.

In the 21 enquiries where there was disagreement, healthcare professionals, on 10 occasions, said the medicine should not be taken, but information from the Helpline was that it was safe to take. In one enquiry the healthcare professional stated the medicine could be taken, whereas Helpline staff recommended the mother to speak again to the physician or a breastfeeding expert about her symptoms, even though the medicine was safe to use. In the remaining 10 enquiries women received conflicting advice from two or more healthcare professionals; in all cases the Helpline staff advised that the medicine could be taken.

For all enquiries where the healthcare professional was either unsure or provided information contradictory to the Helpline, women chose to follow the Helpline advice. Table 4 highlights examples of the enquiries received during the study.

**Table 4:** Examples of enquiries received and answers given

**Enquiry**	**Helpline response**	**Resources used by Helpline**
‘At around 37 weeks pregnant I had assumed that as I had been told to continue my medication (antidepressant) during pregnancy that it would be ok to breastfeed too, but I raised the query with the registrar at my clinic just to double check. He said he thought it would be fine but would check in a book; he did this while I waited and then told me the book said I should not breastfeed. He said he would check with the hospital pharmacy and let me know. I received a letter informing me it was ok for me to breastfeed whilst taking the medication about 4 weeks after my baby was born. Luckily enough I had contacted BfN rather than wait for the hospital to let me know!!’	Information of studies carried out were supplied and an explanation was given that it was important for the patient to continue to take the medication so that she remained well, and that the benefits of breastmilk to her child far outweighed any risk to the baby through her taking medication. The amount of drug passing to the baby was discussed with the mother along with any potential side effects to look out for.	Information was provided using: · Hale· Lactmed (http://toxnet.nlm.nih.gov/cgi-bin/sis/htmlgen?LACT)
‘Got a bad back and wanted to know if it was safe to take naproxen or co-codamol (combination paracetamol/codeine product). Both the doctor and chemist said it was ok to take the medicines’.	The mother was provided with information from Hale on the half lives of the drugs and how long the drug would remain in the mother’s breastmilk. Naproxen has a half life of 12–15 hours and the amount passing into breastmilk is more than other non steroidal anti-inflammatory drugs such as ibuprofen or diclofenac. This makes it less preferable if the baby is under 6 weeks of age before which time hepatic and renal function is lower. There have been concerns raised about the safety of co-codamol during breastfeeding following the death of a baby in Canada. The mother was counselled to watch for signs of unusual drowsiness and poor feeding in the baby. If she noticed these responses she was advised to stop the drug and seek medical advice.	Information was provided using: · Hale · Lactmed (http://toxnet.nlm.nih.gov/cgi-bin/sis/htmlgen?LACT) · http://www.breastfeedingnetwork.org.uk/pdfs/Analgesia_and_breastfeeding_March_2010.pdf http://www.breastfeedingnetwork.org.uk/pdfs/Codeine-in-breastfeeding-June-08.pdf
Have bleeding cracked nipples, slight inversion so using nipple shields. Want something for pain and also want to know what formula to use as concerned about milk supply. Spoke with GP, pharmacist and nurse but none gave a very clear answer.	Discussed moist wound healing and the need to feed or express milk more frequently. Tried to arrange a home visit from a breastfeeding supporter to help with optimising attachment of the baby at the breast, thereby preventing further nipple trauma. Mother was contacted again that evening; she was applying Vaseline to the cracks to prevent scab formation and taking paracetamol. She reported feeling better and will go back to the breast support clinic the following week	Used information from: · BfN leaflet on moist wound healing plus breastfeeding support http://www.breastfeedingnetwork.org.uk/pdfs/Cracked_Nipples_and_Moist_Wound_Healing_2002.pdf
Patient called about general anaesthetic as she was scheduled to have an ovarian cyst removed (planned operation not emergency). Consultant said to stop breastfeeding for 48 hours. Baby was 5 months old.	Mother was informed about short half life of drugs used in general anaesthetic and that some of the drug would remain in fat cells of the body, to be released slowly over the following 48 hours, which might make her baby drowsy. It was suggested that she breastfeed as soon as she felt awake enough to do so. Information on the effect of any anti-emetics, which might be given was also discussed (could increase milk supply). Also discussed how the mother would manage feeds whilst in hospital, and whether she needed to have a breast pump available.	Information was provided using: ·Hale ·Lactmed (http://toxnet.nlm.nih.gov/cgi-bin/sis/htmlgen?LACT) http://www.breastfeedingnetwork.org.uk/pdfs/Day_Surgery_and_Breastfeeding_March_2009.pdf

## Healthcare professional data

Enquiries were taken predominantly from people whose role it is to support new mothers (Table 1). All (100%, n = 24) healthcare professionals believed the Helpline response either completely or partially answered their enquiry, with 92% (n = 22/24) finding the information ‘useful’ or ‘very useful’; consequently, the majority (92%, n = 22/24) would recommend the service to others. Table 2 shows that, in contrast to enquiries from breastfeeding women, the majority (71%, n = 17/24) of healthcare professionals had used the Helpline previously and preferred to email their enquiry (58%, n = 14/24 compared to only 17%, n = 13/77 of women), even though telephone responses were answered more quickly (100% within 24 hours, compared to 79%). Like women, the internet (35%, n = 8/23) and recommendation by another person (26%, n = 6/23) were common ways that they found out about the Helpline, although healthcare professionals also stated leaflets (35%, n = 8/23) were frequently the source of the information.

Analysis of the 24 enquiries showed that 18 were for proposed medicines, 4 involved advice giving, and just 2 were for medicines that were being currently taken (Table 3). Of the 18 medicines for proposed use, 13 (72%) were to treat acute problems. Medicines involved in these cases were wide ranging, with only anti-infectives (n = 2) and domperidone (n = 2) occurring more than once. The 5 enquiries received for proposed medicines to treat chronic conditions were equally diverse, for example medicines to treat schizophrenia, Attention Deficit Hyperactivity Disorder and Cystic Fibrosis. The two enquiries that concerned medicines being currently taken for chronic conditions were montelukast for asthma and levothyroxine for hypothyroidism.

Fourteen (58%) had consulted at least one reference source before contacting the Helpline. The most frequently used reference text was Hale (a specialist text on the safety of drugs in breastfeeding) (n = 9) or the British National Formulary (BNF - a standard text used in the UK for prescribing information) (n = 6). In two cases, a hospital drug information pharmacist was also consulted. Five of the six people that consulted the BNF went on to say that the information it contained was not detailed enough to answer the problem. One of these respondents also stated they did not have access to Hale and thus called the Helpline. Hale was most frequently consulted, yet despite it being more informative than the BNF, 66% of respondents still contacted the Helpline as they believed they would get a more definitive answer based on prior use of the service. Where healthcare staff contacted a specialist drug information pharmacist, the information they provided was deemed insufficient by the enquirer to answer their question.

Four of the 10 healthcare professionals stated that previous positive experiences of the Helpline prompted them to contact the service rather than consult reference sources and two called for reassurance (the other enquirers never specified a reason).

All enquirers said the information provided by the Helpline either completely (n = 20/23, 87%) or partly (n = 3/23, 13%) provided them with enough information to make a decision. Once in possession of the information, 22 went on to say how they used the information. Thirteen passed on the information to the patient, five gave the information to the relevant prescriber and four used it to make a treatment decision.

## Discussion

The findings from this study show that the predominately pharmacist-led service provided by the BfN is well received by both healthcare professionals and breastfeeding women, and is consistent with user satisfaction surveys of pharmacist-led drug information services [20,21]. Unlike most information services, the BfN Helpline actively encourages the public to contact the service, and this was reflected in a higher proportion of enquiries taken from the public compared to healthcare professionals. Most women were previous non-users of the service in contrast to healthcare professionals, where the majority had contacted the service before. The healthcare professionals predominantly had a specialist role associated with maternal services and this may explain why they were aware of the service.

Provision of the Helpline seems well-founded given the level of conflicting information given by two or even three healthcare staff, and the lack of confidence or distrust women had in answers they provided. The disparity seen between the answers given by the Helpline and the responses from healthcare staff further reinforces the decision by their patients to seek out specialist advice. The fact that over 75% of healthcare staff did not know the answer, or gave a conflicting answer compared to that given by Helpline staff, suggests that healthcare staff may not have sufficient knowledge or resources in this field; which echoes previous findings [2-7,29,30]. Where Helpline staff contradicted healthcare staff advice, breastfeeding women chose to follow Helpline advice. This was not unexpected as many women had contacted the Helpline as a ‘last resort’. However, in clinical practice the contradiction of one healthcare professional by another can be difficult, especially when one is perceived as more authoritative than the other (e.g. lactation consultant doctor versus a pharmacist). In these situations, it would seem most prudent for the contradicting person to talk with the person who gave the initial advice, so that a consistent message is given to the breastfeeding women. This did not routinely appear to happen in this study.

Findings from this study also raise questions about the suitability of ‘generalist’ healthcare staff to handle breastfeeding enquiries; in particular, physicians, as they were most consulted. A study by Wallace and Kosmala-Anderson reinforces this view, as they found that general practitioners in the UK saw updates on prescribing for breastfeeding mothers as a low priority, with just 19% highlighting this as an area of need, despite only 34% describing themselves as competent or expert in this field [31].

Only just over half of healthcare professionals had consulted a reference source, and those that did found standard texts, such as the BNF (which almost all UK healthcare staff have easy access to), to be inadequate, as data contained within it lacked sufficient detail. The deficiency of the BNF has been highlighted by the National Institute for Health and Clinical Excellence (NICE) and advocates that healthcare professionals consult supplementary sources of information [32]. Whilst some healthcare staff did consult other sources (i.e. the specialist text by Hale), they still felt unsure on how to interpret the information in a way that was useful to the mother, and sought guidance from the Helpline. This lack of confidence in interpretation of appropriate reference sources suggests a greater need for training. It further supports the use of a specialist, given their access to more resources (as evidenced from Table 4) and ability to interpret the data to provide informed answers on medicine suitability.

A study by Akus also found that healthcare practitioners were using outdated sources for making safety recommendations to their patients [33]. Whilst this cannot be substantiated in this study, other studies [34] have found similar results to Akus and it seems unlikely that healthcare staff in practice are using the most up-to-date reference sources when evaluating medicine suitability for breastfeeding mothers.

Enquiries predominantly related to proposed medicines to treat acute self-limiting problems. Advice given by healthcare staff to patients erred on the side of caution; that is they were told to avoid taking the medicine. Whilst this negates any possible adverse events to the child, it does not necessarily equate to appropriate clinical care where decisions should be evidence-based and an assessment of the risk versus benefits to the baby made [35,36]. In defence of healthcare staff, the lack of manufacturer data on the effects of medicines passing in to breastmilk makes recommendation of products problematic. Without safety data, manufacturers generally advocate avoidance. If products are then recommended, prescribers do so outside of the product licence, and liability will fall on that individual and not the company. Amir and colleagues looked at knowledge, attitude and practices of Australian GPs in obtaining information for medicines for breastfeeding women, and found that medico-legal concerns were common, and 76% stated that this was important in their decision-making [37].

What is not in question is that patients will continue to require medication during breastfeeding. Studies have shown that this is relatively commonplace [38-40], and medicines play an important part in a woman’s decision to start and continue breastfeeding, with women frequently hesitant to combine the two, choosing either to stop breastfeeding or attempt to limit exposure [40-42]. Therefore, given healthcare professional uncertainty and caution in advice-giving, it was interesting to note that the Helpline was able to give definitive answers in almost all cases relating to medicine taking. Given that other countries do experience similar problems with under-skilled healthcare staff in this field, then the establishment of similar Helplines could be considered. The costs of running the BfN Helpline was initially modest, but as call numbers have increased the Helpline has had to rely on greater levels of goodwill from the people that answer the enquiries. From our experience it would be prudent, when starting such a Helpline, to factor in additional resources and money to meet increased demand.

## Limitations

It is acknowledged that this study provides a ‘snapshot’ of the service, and the number of participants was relatively low. In addition, the study had a high drop-out rate/lost to follow-up, which may have affected the data through non-response bias. Lastly, the findings may not be broadly generalisable to other countries due to differing healthcare structures.

## Conclusion

The Breastfeeding Networks’ Drugs in Breastmilk Helpline provides an important service to patients and healthcare staff to make informed decisions on medicine-taking whilst breastfeeding. The relatively high level of healthcare professional uncertainty or incorrect advice given to patients, coupled with patient distrust, signals that healthcare professional education needs improving and that greater use of specialist services should be encouraged.

## Competing interests

The work was supported by the Breastfeeding Network to employ Miss Seera and Miss Matharu to conduct the telephone interviews. Wendy Jones is employed by the Breastfeeding Network to answer calls, but was not involved in data collection or analysis (except for agreeing qualitative themes).

## Authors’ contributions

PR and WJ constructed the data collection tool. WJ answered the majority of the calls. PR oversaw the telephone interviews and verified data capture. PR undertook the analysis. PR and WJ jointly wrote the manuscript.
